# New approaches to uncover COPD pathobiology and develop therapies

**DOI:** 10.1172/jci.insight.199693

**Published:** 2026-02-23

**Authors:** Yohannes Tesfaigzi, Ali Önder Yildirim, Francesca Polverino, Thomas M. Conlon, Venkataramana Sidhaye, Maor Sauler, S. Vamsee Raju, Renata Z. Jurkowska, Divay Chandra, Michael H. Cho, Edwin K. Silverman, Ramon C. Sun, Peter Castaldi, Purushothama Rao Tata, Kambez H. Benam, Linto Antony, Mareike Lehmann, Beata Kosmider, Karim Bahmed, Zerihun H. Negasi, Kamakshi Bankoti, Carter Swaby, Dave A. Lagowala, Yeşim Vural, Hasan Bayram, Rosa Faner, George Washko, Dinh Son Bui, Bartolome Celli, Roxana Maria Wasnick, Enid Neptune

**Affiliations:** 1 Division of Pulmonary and Critical Care Medicine, Department of Medicine, Mass General Brigham and Harvard Medical School, Boston, Massachusetts, USA.; 2 Institute of Lung Health and Immunity (LHI), Helmholtz Munich, Comprehensive Pneumology Center (CPC-M), Member of the German Center for Lung Research (DZL), Munich, Germany.; 3 Institute of Experimental Pneumology, LMU University Hospital, Ludwig-Maximilian’s University, Munich, Germany.; 4 Koc University Research Center for Translational Medicine (KUTTAM), Istanbul, Turkey.; 5 Pulmonary and Critical Care Medicine, Baylor College of Medicine, Houston, Texas, USA.; 6 Department of Medicine, Division of Pulmonary and Critical Care Medicine, Johns Hopkins University School of Medicine, Baltimore, Maryland, USA.; 7 Division of Pulmonary, Critical Care, and Sleep Medicine, Yale School of Medicine, New Haven, Connecticut, USA.; 8 Department of Medicine, Division of Pulmonary, Allergy and Critical Care Medicine, The University of Alabama at Birmingham, Birmingham, Alabama, USA.; 9 Division of Biomedicine, School of Biosciences, Cardiff University, Cardiff, United Kingdom.; 10 Division of Pulmonary, Allergy, Sleep and Critical Care Medicine, Department of Medicine, University of Pittsburgh, Pittsburgh, Pennsylvania, USA.; 11 Channing Division of Network Medicine, Brigham and Women’s Hospital and Harvard Medical School, Boston, Massachusetts, USA.; 12 Department of Biochemistry and Molecular Biology, College of Medicine, University of Florida, Gainesville, Florida, USA.; 13 Departments of Cell Biology and Medicine, Duke University School of Medicine, Durham, North Carolina, USA.; 14 Department of Medicine, Bioengineering, University of Pittsburgh, Pittsburgh, Pennsylvania, USA.; 15 Lung Aging and Regeneration, Institute for Lung Health (ILH), Giessen, Germany.; 16 Institute for Lung Research, Philipps-University Marburg, Universities of Giessen and Marburg Lung Center, German Center for Lung Research (DZL), Marburg, Germany.; 17 Department of Microbiology, Immunology and Inflammation, Center for Inflammation and Lung Research, Temple University, Philadelphia, Pennsylvania, USA.; 18 Department of Cell Biology, and; 19 Department of Environmental Health and Engineering, Johns Hopkins University School of Medicine, Baltimore, Maryland, USA.; 20 Department of Cellular and Molecular Medicine, Graduate School of Health Sciences, and; 21 Department of Pulmonary Medicine, School of Medicine, Koc University, Istanbul, Turkey.; 22 Biomedicine Department, University of Barcelona, IDIBAPS, CIBER, Barcelona, Spain.; 23 Center for Epidemiology and Biostatistics, University of Melbourne, Melbourne, Victoria, Australia.

## Abstract

Chronic obstructive pulmonary disease (COPD) was the third leading cause of global mortality in 2011 but receives limited attention and research funding. This Review describes the current knowledge on COPD risk factors, including genetic and epigenetic determinants and their interactions with the microbiome and environmental exposures. Preclinical models are being refined and single-cell transcriptomic, metabolomic, and proteomic technologies are being implemented to investigate the molecular mechanisms of disease progression. Patient cohorts to define biomarkers of early disease and the latest approaches to diagnose pre-COPD are essential to accelerate the development of novel and effective therapeutic interventions and translate new findings into clinical trials. This Review is a summary of topics covered by a symposium organized by the COPD-iNET consortium, an international network of researchers who have established a platform that facilitates collaboration of this multidisciplinary group of preclinical, translational, and clinical researchers.

## The worldwide burden of chronic obstructive pulmonary disease

Worldwide, chronic obstructive pulmonary disease (COPD) affects approximately 400–600 million people, significantly strains health care systems (1), and is the third leading cause of death (2–4). What is less emphasized is that the definition of COPD as a clinical disorder has evolved. Beyond tobacco exposure, the recognition of early life events, distinct permissive or directive immune responses to environmental triggers, and a staged evolution in the disorder reflects nuances delivered by the intensive clinical translational and basic research efforts pursued over the past 25 years. Despite the profound disease burden, compared with other less common lung diseases, COPD research has historically received limited attention and resources (5, 6).

What is achievable for COPD in 2026? Efficient, integrative, and respectful use of advanced in vitro and in vivo testing modalities informed by in silico datasets cohere into a standard of team science providing mechanistic validation for therapeutic targets. Candidate therapeutics are fueled by promising delivery systems and highly targeted agents, such as nanobodies, customized vectors, small molecules with defined spectra of activity, and repurposed pharmacologics capable of accelerated approval for use. Clinical trials can adopt the creativity of COVID-era studies that incorporate multiple agents, shorter time scales, and both surrogate and definitive readouts. The cures are ready for launch. This Review will focus on the current frontier of COPD investigations that have the potential to transform COPD research.

**Figure 1:**
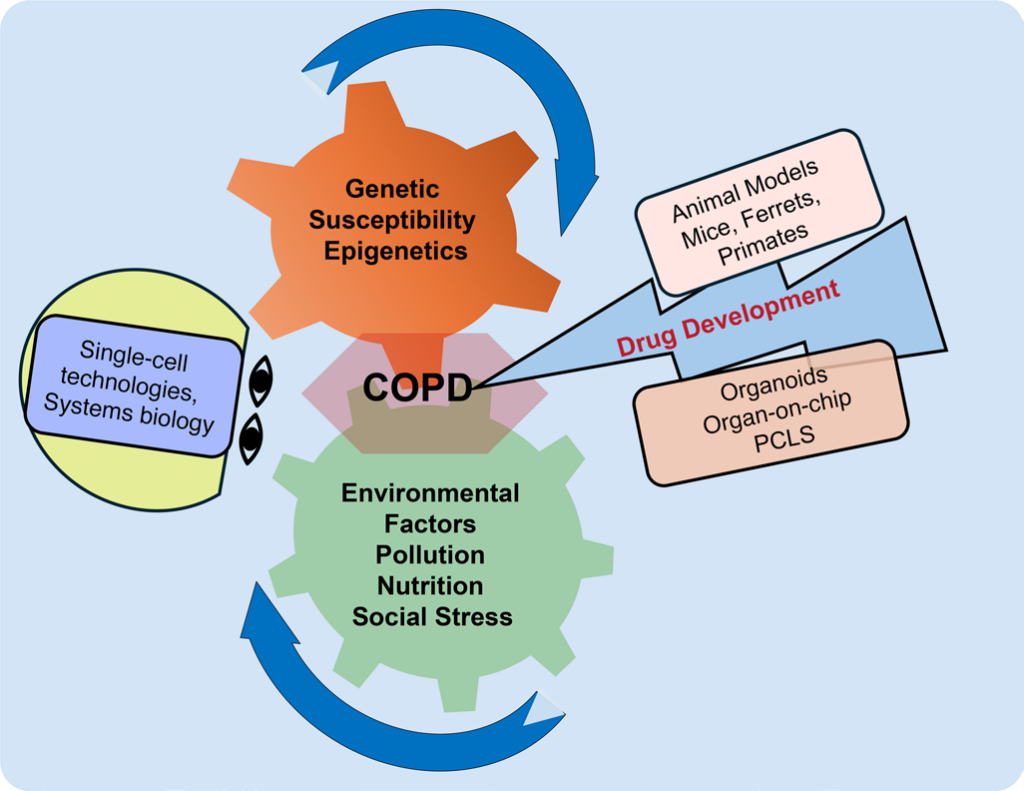
COPD-iNET symposium focused on five key areas critical to understanding the mechanistic underpinnings of COPD. Genetic and environmental factors drive each other to cause disease progression. Cohorts with early-stage COPD help unravel the complex interactions leading to disease. Advances in single-cell technologies offer detailed insights into cellular changes. At the same time, improved preclinical models support the development and screening of therapeutic candidates capable of interrupting the cycle of gene-and-environment interaction to halt COPD progression.

Why this, why now? In response to the great need to accelerate progress in COPD research, an international network of basic and translational researchers has formed the COPD-iNET consortium to facilitate knowledge exchange and form new research collaborations (7). The second annual COPD-iNET symposium brought together leading experts in the field to explore and discuss the latest findings, align research priorities, and integrate findings toward developing reliable diagnostics and treatments for COPD. The symposium, held in Boston, Massachusetts, was generously supported by the American Lung Association (ALA). Over 115 participants gathered in person to address five key COPD research areas (Figure 1) — genetics, environmental factors, model systems, drug development, and new technologies. The goal of COPD-iNET is to provide a platform that facilitates multidisciplinary research involving basic, translational, and clinical data to be harmonized for meta-analyses. This platform will accelerate the drug development pipeline and increase the success rate of efficacy in COPD subtypes. This Review summarizes key insights across these domains and outlines key challenges and future directions to study the molecular mechanisms and development of therapies for COPD. Certain areas of interest in COPD are beyond the scope of this Review and are best served by more intensive and detailed articles.

## COPD definition and subtypes

In the late 1990s and early 2000s, COPD classification was significantly transformed due to efforts of the American Thoracic Society and the European Respiratory Society, who published the first guidelines aimed at improving COPD diagnosis, classification, and management (8). Next, the Global Initiative for Chronic Obstructive Lung Disease (GOLD) established worldwide recommendations to standardize COPD diagnosis and management (9). Together, with the classical 4 stages (stage 1–4) of spirometrically recognized airflow obstruction severity, GOLD introduced the concept of “GOLD stages,” with stage 0 defined as individuals experiencing chronic respiratory symptoms, but with normal spirometry. Abnormal spirometry is defined by a ratio of forced expiratory volume in one second to forced vital capacity (FEV_1_/FVC) of less than 0.7 and severity defined by reduced FEV_1_ (10) (Table 1).

Clinically, COPD is a progressive disorder characterized by largely irreversible airflow limitation. COPD encompasses two major histological variants: emphysema, characterized by alveolar destruction; and chronic bronchitis, defined by mucus hypersecretion, chronic inflammation, airway wall thickening, and alveolar remodeling, all of which reduce quality of life (11). Most patients have a combination of these manifestations, and disease heterogeneity creates a challenge to classify COPD into distinct subtypes. Recently dupilumab, an IL-4/IL-13 receptor–targeting mAb, reduced exacerbations and improved lung function (12) in patients with COPD who also had a high eosinophil count; however, this success is limited to a small percentage of patients. The advancement of disease-modifying therapies for COPD is still required, as the mortality rate remains high.

## “Pre-COPD” as an entity and available cohorts

Observational studies of long-term smokers consistently show a constellation of respiratory symptomatology that precedes obstructive lung physiology (13). The “pre-COPD” concept emerged (14) for certain clinical respiratory symptoms and physiological and imaging abnormalities in a heterogeneous group of individuals at high risk of developing clinical overt COPD (15). Early and pre-COPD risk factors often overlap and are based on their strong links to early lung function decline and disease progression and highlights a growing recognition that COPD develops long before spirometric obstruction and clinical symptoms become apparent (16, 17).

**Figure 2:**
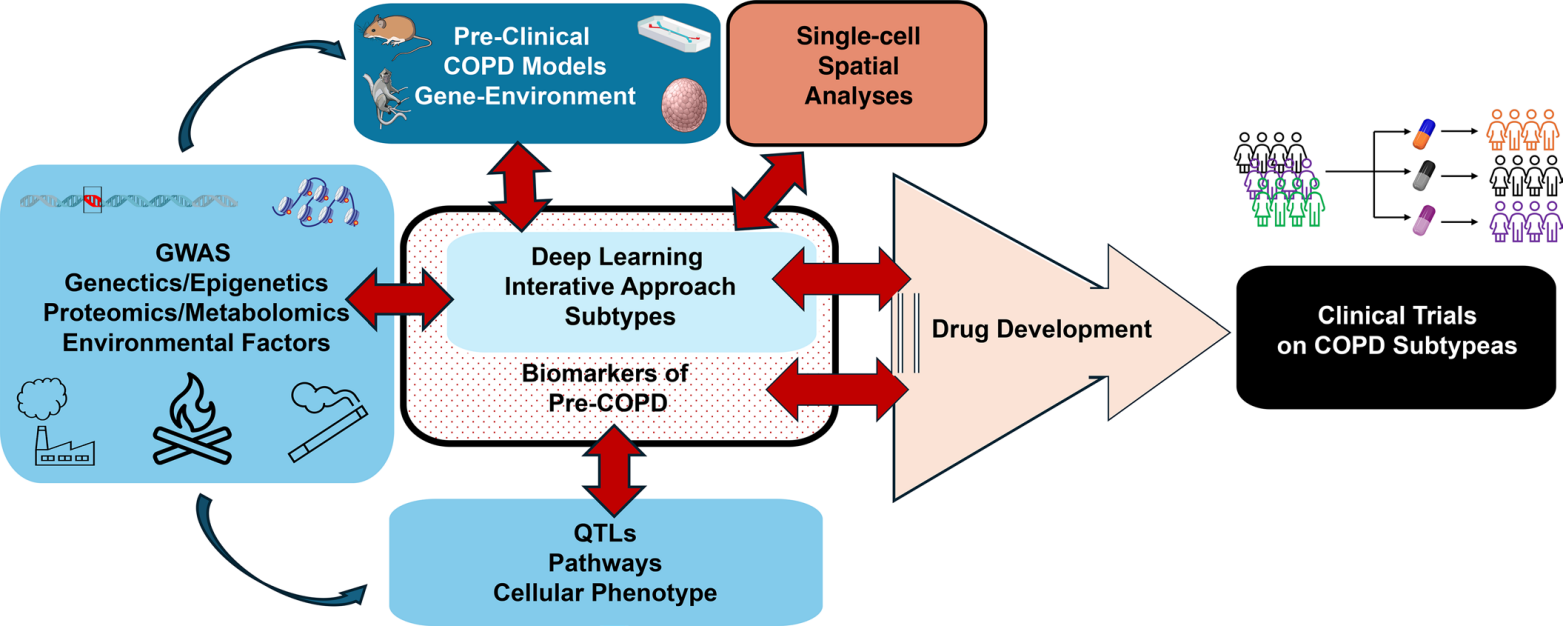
Evolving landscape of COPD research. GWAS provide information on susceptibility genes and DNA methylation. ChIP-seq and ATAC-seq from appropriate tissues provide epigenetic modifications that are associated with environmental factors. Data from specific tissues and cell types generate QTL information that points to genes and reliable information on pathways involved in affecting cellular phenotypes and pathways that could be initiators of disease. This in turn can be used to develop appropriate preclinical COPD models that capture the gene-and-environment interaction. Applying deep learning algorithms to scRNA-seq and spatial RNA-seq data from preclinical and pre-COPD cohort samples, together with the GWAS and QTL data, can be used to understand COPD heterogeneity. An iterative process of data analysis and retesting in experimental models and findings from pre-COPD cohorts helps to prioritize for testing appropriate drugs that will be successful in clinical trials and have minimal side effects.

Several methods are now established and used to define changes in the lung to detect pre-COPD. Oscillometry requires minimal patient cooperation, making it particularly well suited for pediatric use (18). Computerized CT scans identify structural abnormalities and CT remains one of the most potent tools in the early detection of pre-COPD (19). MRI offers a radiation-free approach that is exceptionally sensitive to soft-tissue changes. Hyperpolarized helium or xenon MRI can detect lung ventilation abnormalities, even when CT scans and spirometry appear normal (20). Analyses of electronic health records, imaging data, and wearable device data may allow continuous monitoring in real-time and help clinicians to predict which individuals at risk to progress to COPD (21–23) (Figure 2).

A landmark study analyzed lung function trajectories of individuals between ages 25 and 75 from three independent cohorts — the Framingham Offspring Cohort, the Copenhagen City Heart Study, and the Lovelace Smokers Cohort. While some individuals reached 100% predicted FEV_1_ in early adulthood, others only reached 75%, with both groups showing either stable or rapidly declining lung function (24). Those with low baseline lung function and rapid decline had a 35-fold higher risk of developing COPD within 18 months and a 2.5-fold higher mortality risk compared with those with higher lung function (16). In the CARDIA Lung Study, longitudinal lung function trajectories were linked to COPD development or pre-COPD (25). Preserved ratio impaired spirometry (PRISm) — defined as a reduced FEV_1_ with preserved FEV_1_/FVC ratio (26) — is prevalent (13% of the COPDGene cohort and 7.1% in the general population of adults >45 years of age) and associated with increased respiratory symptoms, systemic inflammation, and a cardiovascular mortality rate of 18.7% (27).

ALA launched the Lung Health Cohort, a new, population-based study focused on characterizing lung health in millennials (ages 25–35) without diagnosed severe respiratory disease (28). The Ghana randomized air pollution and health study (GRAPHS) enrolled 1,414 nonsmoking pregnant women and has followed their children for over eight years. Early findings show that high prenatal exposure to household air pollution is linked to impaired lung function in infancy and early childhood (29). The Tasmanian longitudinal health study (TAHS) identified three of six trajectories with increased COPD risk as measured at 7, 13, 18, 45, 50, and 53 years of age (30, 31).

The Subpopulations and Intermediate Outcome Measures in COPD Study (SPIROMICS) cohort showed that elevated MUC5AC, but not MUC5B, in sputum is a biomarker for pre-COPD. Genetic and proteomic studies continue to explore reliable biomarkers to identify pre-COPD (32–34). Inflammatory markers in blood (35), sputum (36), and exhaled breath condensate have shown promise for detecting early inflammatory changes prior to spirometric decline. Notably, lower circulating CC16 levels and higher MMP levels have been associated with early lung function impairment. In the COPDGene study, both low CC16 (37) and soluble RAGE (38) were linked to increased risk of disease progression and poor outcomes. In COPDGene, individuals with high polygenic risk score (PRS) were more likely to exhibit early airflow limitations, even in the absence of spirometrically defined COPD (39, 40).

## COPD-associated risk factors

Infants born prematurely with underdeveloped lungs have an increased COPD risk. These infants may experience reduced lung growth, airway obstruction, and impaired lung function that persist into childhood and adulthood (30, 41, 42) (Figure 2). Furthermore, frequent or severe respiratory tract infections in childhood, including influenza, respiratory syncytial virus (RSV), and bacterial infections, that cause bronchitis, pneumonia, and recurrent wheezing episodes can cause lasting damage to developing airways and lungs, leading to changes that prevent normal lung growth and accelerate age-related lung function decline, especially when combined with other risk factors, such as smoking or other environmental exposures (30, 41, 42). Childhood asthma has also been linked to developing early COPD, as persistent airway inflammation and recurrent obstruction can lead to long-term lung damage, especially when combined with other risk factors (42, 43).

This Review will primarily focus on the most recent concepts in COPD-related genetic risk factors, epigenetics, and gene-environment interactions, as these topics have previously been well covered (44–46).

### COPD susceptibility genes.

The most widely described genetic factor contributing to COPD development, particularly emphysema, is α-1 antitrypsin deficiency (AATD), a hereditary condition first described in 1963 (47). AATD is usually caused by homozygosity at the SNP rs28929474 in serpin family A member 1 (*SERPINA1*), which encodes a missense variant of the AAT protein. Other loss-of-function alleles, including rare nonsense variants, can exist as homozygotes or complex heterozygotes and associate with COPD. AAT primarily functions to inactivate neutrophil elastase (48). The prevalence of severe AATD is estimated at 1%–2% of the population (49). As with other susceptibilities, smoking increases the risk of COPD in AATD, and this exacerbation is an early example of gene-by-environment interaction (50).

GWAS has successfully identified thousands of genetic risk loci for human traits and chronic diseases (51) by scanning the entire genome of many individuals to find genetic differences (typically SNPs) that correlate with a particular condition or phenotype. For COPD, GWAS has identified SNPs in or near *HHIP*, *FAM13A*, and *MFAP2* (52–54), and numerous genome-wide loci associated with FEV_1_/FVC ratio and FEV_1_ have been identified (55, 56) (Figure 2).

While the contribution of genetic variants to COPD development is well established, functional validation remains challenging. First, many analyses have focused on cohorts of patients with late-stage COPD, a point by which it is unclear what effect a genetic variant had on disease initiation in the early stages and what is the result of secondary damage at later stages. Because patients at early COPD stages do not present with respiratory symptoms, it is difficult to obtain cells initially affected by the variant. Second, GWAS results alone usually do not provide definitive information on functional mechanisms. Third, determination of the genes affected by GWAS loci (target genes) can be challenging, as most of the causal variants are located in noncoding sections of genome (57) (intergenic or intragenic regions) (58), indicating that these loci may regulate gene expression via complex interactions with distal regulatory elements. A molecular understanding of causative SNPs has been challenging, because a single SNP or multiple SNPs can affect one or multiple cell types, and each can amplify the response to pollutants and the resulting cellular interactions in the lung (59).

The ability to combine multiple genetic variants into a PRS can generate a numerical value that reflects an individual’s predictive risk of developing COPD independent of clinical risk factors (40, 60). Although powerful, genetic predisposition alone is not enough; interactions between genes and environmental factors must be considered. Indeed, integrating information from inherited (genetic) and environmental (epigenetic) DNA and chromatin changes can point to key genes and pathways associated with COPD development and progression (Figure 2).

Additional -omics approaches can also be integrated with genetics to investigate the molecular heterogeneity in COPD. For example, integration of transcriptomics, DNA methylation, and miRNA profiles was recently used to identify disease endotypes associated with clinical COPD characteristics, such as FEV_1_ or blood eosinophils (61). When undertaking such approaches it is important to consider that unsupervised clustering across cohorts to identify COPD subtypes may show modest reproducibility (62): however, trajectory analyses are more promising, as they integrate additional factors such as longitudinal lung function (63).

A successful strategy to identify GWAS target genes is to combine GWAS and expression quantitative trait locus (eQTL) studies, which test the association between genetic variants and RNA levels of nearby genes (Figure 2). These combined analyses identify genomic regions where GWAS and eQTL association signals coincide, and colocalization methods apply statistical analyses that distinguish causal from chance or correlational (linkage disequilbrium) GWAS-eQTL overlap by estimating the probability that both signals are caused by the same functional variant(s). QTLs at single-cell resolution could provide insight into molecular mechanisms underlying the pathobiology of COPD by exploring the interface of altered gene expression and the resulting biological consequence (64).

### Environmental factors.

**Figure 3:**
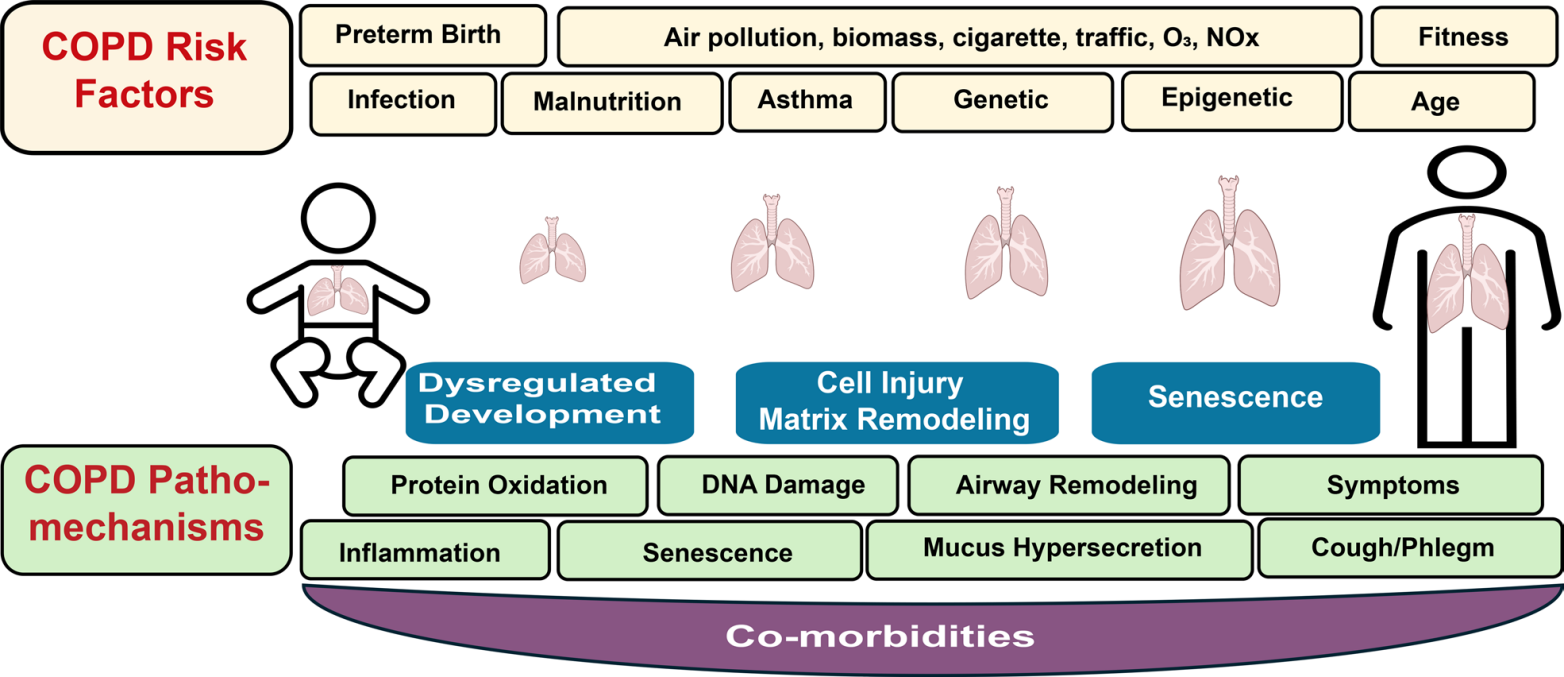
Risk factors and pathomechanisms driving COPD development. Starting from the early stages of embryonic development throughout growth into adulthood, the lung is exposed to many risk factors. Many of these risk factors can affect individuals to different extents and in different combinations, as do the many pathomechanisms that comprise a wide range of cellular pathways. More importantly, these pathomechanisms are also affected by comorbidities that enhance disease progression.

COPD-associated genetic factors likely interact with environmental factors, such as smoking, occupational exposures, and air pollution, to cause disease. However, research on the cellular pathways and mechanisms that link environmental factors to genetic variants to drive disease is in its infancy. It is of great importance to understand the mechanisms underlying environmental factors that drive COPD development to ultimately develop therapies. Studies on the effects of different types of particulate matter (PM) derived from air pollution, cigarette smoke (CS), wood smoke (WS), and gases, including ozone (O_3_) and nitrogen oxides on cellular systems is complex. In addition, how societal stress conditions and fitness interact with genetic and epigenetic susceptibility factors have not been explored (Figure 3).

PM is a category of inhalable particles classified by size rather than chemical composition. PM is a complex mixture from both natural sources, such as vegetation and dust, and combustion emissions from cooking or heating stoves, wildfires, and other human activities, including cigarette smoking and industrial emissions (65). Localization of these particles in lung tissue largely depends on size — particles larger than 10 μm (PM_10_) typically settle in the nose and throat, those between 2.5 and 10 μm (PM_2.5–10_) deposit in larger airways, particles less than 2.5 μm (PM_2.5_) reach lower airways, and nanoparticles (<0.1 micrometers, PM_0.1_) can be internalized into the alveoli (66). While in high-income countries, PM from CS is the primary cause for COPD, in both high- and low-income countries, PM from biomass smoke from cooking or heating stoves, dust storms, traffic, and wildfires contribute to the onset and progression of COPD (67, 68),

Long-term exposure to even low air pollutant levels can increase COPD incidence (69). The Framingham Heart Study showed a correlation between PM_2.5_ exposure and reduced lung function metrics like FEV_1_ and FVC (70). PM_2.5_ reduces FEV_1_, increases COPD risk (71), and caused lung function decline both in the Lovelace Smokers Cohort (72) and the SPIROMICS cohort (73). Additionally, a retrospective Korean cohort study found a significant association between long-term PM_2.5_ exposure and COPD development (66). Table 2 summarizes environmental factors, their main sources, and roles in COPD development. Gaseous pollutants, including nitrogen dioxide (NO_2_), O_3_, and sulfur dioxide (SO_2_), primarily originate from fossil fuel combustion.

Exposure of cells to pollution and the inflammatory milieu enhances oxidative damage, promoting cellular apoptosis, senescence, and destruction of alveolar walls. Airway remodeling is the persistent change in lung structures due to repeated epithelial damage, including goblet cell hyperplasia, which causes persistent and increased mucus production. Furthermore, inflammatory stressors can cause extensive changes to genomic structures, including epigenetic reprogramming of many lung cells, such as the airway epithelium, fibroblasts, and inflammatory cells. Importantly, small airway pathologies, such as narrowing of airways and thickening of airway walls due to smooth muscle hypertrophy and fibrosis, are thought to occur before the loss of alveolar tissue and lung function decline (74).

Populations from low- and high-income countries are exposed to biomass fuel smoke from open wood or dung fires, stoves to cook or heat homes, and from recent increased occurrences of wildfires (75, 76). While some wildfire pollutants are short-lived and disperse quickly from the fire site, PM can travel vast distances, far exceeding the size of the original fire area (77). Dust storms from the Middle East and Sahara Desert are associated with an increased hospitalization risk for patients with COPD (78).

Wildfires also have adverse effects on birth weight, respiratory, cardiovascular, and other diseases (79, 80). Systematic meta-analyses concluded that pollution from WS not only increases the risk for COPD exacerbations (81, 82) and pneumonia (83), but more than doubles the risk of COPD and chronic bronchitis (84–86), and increases emergency room visits, hospitalizations, and mortality (87–89). Coarse PM (PM_2.5–10_) extracted from the 2008 California wildfires rapidly induced cell death in murine macrophages in vitro, with significantly higher cytotoxicity than coarse PM collected from ambient air (90). Similar rapid cytotoxic effects on alveolar macrophages were observed using bronchoalveolar lavage in a murine model following intratracheal instillation of wildfire coarse PM (91).

Daily or occasional E-cigarette users have higher odds of developing COPD compared with non-users (92). A long-term cohort study of 10,326 Chinese adults reinforced this risk, with a significant correlation between E-cigarette use and respiratory symptoms or COPD, especially with concurrent combustible cigarette use (93). Waterpipe exposure elicits an inflammatory response similar to that of conventional smoking (94), and in vitro studies indicate that it causes epithelial barrier dysfunction and reduces lung cell proliferation, while increasing markers of cellular senescence (95, 96).

### Combined effects of ambient air pollution on lung health.

Population studies have demonstrated that PM_2.5_ from CS and WS have an additive effect on reducing lung function and risk for COPD (71), an effect that has also been validated in animal studies (97, 98). Therefore, stratifying PM_2.5_ into specific, relevant environmental exposures such as tobacco and wildfire smoke may be important to fully understand the effect of various sources of PM_2.5_.

## Gene-by-environment interaction

### Environmental exposures drive epigenetic changes.

CS, PM, and biomass fuels have all been shown to induce profound epigenetic modifications (99, 100) that facilitate interactions between environmental factors and gene expression. DNA methylation and histone modifications modulate transcriptional activity without altering the underlying DNA sequence (101). Treatment with global demethylating agent 5-azacytidine mitigates CS-induced alveolar destruction in human precision-cut lung slices (PCLS) (102). Emerging evidence also suggests that DNA methylation may provide a sensitive biomarker for COPD detection and patient stratification (103, 104).

DNA methylation, which is catalyzed by DNA methyltransferases, is regulated by various establishment and maintenance enzymes (105). The ten-eleven translocation (TET) family of proteins facilitates DNA demethylation (106). Notably, TET1 and TET2 are reduced in response to CS and are low in COPD (107). In contrast, DNMT1, which maintains DNA methylation, and DNMT3a, responsible for de novo methylation (108), are elevated in lung tissues of patients with COPD, with DNMT3a showing a strong negative correlation with pulmonary function (109). While DNA methylation is primarily an epigenetic modification, the chemical structure of 5-methylcytosine (5mC) increases spontaneous deamination, leading to a CG-to-TG transition (110), often triggered by oxidative stress, a common consequence of environmental exposures. This suggests that even methylation can result in genetic changes (Figure 3).

Histone acetylation, catalyzed by histone acetyltransferases and removed by histone deacetylases, typically enhances DNA accessibility and promotes gene transcription. These proteins have been broadly linked to inflammation, corticosteroid resistance, and changes in epithelial barrier integrity and contribute to COPD (111, 112). Furthermore, histone methylation can alter chromatin accessibility and change gene activation. For instance, the histone methylation mark H3K4me3 is associated with open chromatin and active transcription, whereas H3K27me3 and H3K9me3 marks are associated with closed chromatin and inactive transcription (113, 114). These broad mechanisms may not adequately capture the complexity and variability within biological systems, highlighting the need for a more nuanced understanding of the factors influencing epigenetic responses. Several genome-wide studies, including recent high-resolution whole-genome bisulfite sequencing (WGBS) studies in purified lung cells support widespread DNA methylation changes in COPD (105).

Epigenetic profiling of chromatin accessibility, 3D interactions, DNA methylation, enhancer RNA, and specific histone modifications may help identify which variants affect gene expression in specific cell types. These approaches have potential to identify risk variants by providing functional annotation across the genome. Indeed, recent high-resolution data from purified lung cells indicate that DNA methylation changes in COPD cells often occur in regulatory regions, including enhancers (104). An additional potential mediator of genetic risk are short noncoding miRNAs (115, 116), which offer an exciting therapeutic potential due to their ability to transfer messages via extracellular vesicles (EVs) from cell to cell and propagate disease. Indeed, exciting data this year revealed that COPD airway epithelial cell–derived EVs enriched with miR-34a could spread cellular senescence to neighboring cells, potentially accounting for disease progression (117). The integration of genetic and epigenetic analyses may provide functional mechanisms linking genetic variants to chronicity of disease pathology.

### Microbiome changes affected by environmental exposures.

Environmental factors significantly influence the composition and diversity of the lung microbiome. Toxic exposures, including CS and air pollution, can disrupt the delicate balance of microbial communities in the pulmonary system, leading to dysbiosis. For instance, CS contains a wide array of harmful chemicals that can alter microbial populations and promote an inflammatory environment within the lungs (118).

Patients with moderate to severe COPD often show greater enrichment of oral bacteria in the lung (119), potentially due to swallowing difficulties and gastroesophageal reflux, which may allow oral bacteria to colonize the lower airways (120). Impaired mucociliary clearance in COPD can further contribute to microbiome dysbiosis (121). Additionally, dysbiosis in the oral cavity may impact airway health. A systematic review highlighted a correlation between periodontal disease and COPD, thus improving oral health may mitigate respiratory disease progression, especially in high-risk elderly patients (122).

## Advances in preclinical COPD models

Effective drug development requires laboratory models that closely recapitulate the complexity of multifactorial etiology and patient heterogeneity to increase the likelihood for effective clinical translation (123). Table 3 highlights current and emerging preclinical models, including in vitro/ex vivo and animal models for COPD research and lists their strengths and weaknesses.

## In vitro and ex vivo models

Air-liquid interface cultures allow basal cells to differentiate into a pseudostratified mucociliary epithelium (124). Organs-on-chip, also known as microphysiological systems (MPS), are biomimetic devices that incorporate living cells within perfusable microfluidic channels and/or chambers (125–128). These systems replicate key features of human organs by recreating multicellular architectures, tissue-tissue interfaces, biochemical gradients, mechanical forces, and dynamic vascular perfusion. Multiple human lung MPS have been developed to model distinct lung regions, including small airways, alveoli, parenchymal microvasculature, and stroma (125, 129, 130). These models reproduce hallmark features, including smoke-induced oxidative stress, inflammation, and epithelial barrier disruption, while also enabling the evaluation of therapeutics and precision medicine strategies (131).

PCLS provide a valuable ex vivo approach that preserves native tissue architecture and cellular diversity of the diseased lung. PCLS are typically prepared by filling lungs with agarose and cutting into thin (100–500 μm) slices, thus maintaining viable cells in their original spatial relationships, including epithelial-mesenchymal interactions and intact microvasculature (132–134). PCLS derived from patients with COPD or animal models facilitate direct translational comparisons, potentially improving predictive validity (135). This model permits detailed investigation of regional heterogeneity, a critical COPD aspect of not easily addressed in isolated cell cultures (136).

Lung organoids are 3D structures derived from stem/progenitor cells that self-organize to recapitulate aspects of lung development and architecture. Organoids bridge the gap between traditional cell culture systems and animal models, providing intermediate complexity with enhanced physiological relevance (137). Organoids have been successfully used to identify novel regenerative therapies in COPD (138). COPD-derived organoids revealed a role for dose-dependent hedgehog-mediated signaling in proximal-distal identity of fibroblast maintenance and the effect of its dysregulation on alveolar regeneration and emphysema (139). In particular, induced pluripotent stem cell–derived (iPSC-derived) organoids have been instrumental in modeling the effect of GWAS-identified COPD genes on modulating alveolar type 2 (AT2) pneumocyte cell function (140). Future studies need to expand tissue engineering approaches to reconstruct anatomical sites, like respiratory bronchiolar cells, that currently lack well-established genetic models (141).

In summary, in vitro and ex vivo enable detailed mechanistic studies under controlled conditions, providing high-throughput screening platforms and enhancing translation through direct investigation of human tissues.

## Animal COPD models

Mice have dominated preclinical COPD research due to their well-characterized genetics, strain availability, lower maintenance costs, smaller size, and shorter lifespan. Genetic factors across murine species influence susceptibility to CS-induced emphysema. In a screen of 34 inbred mouse strains, A/J mice were most susceptible and CBA/J mice were least vulnerable due to an *Abi3bp* variant (142). Compared with male C57BL/6 mice, female animals are resistant to CS-induced emphysema, while these sex-specific differences are not observed in C3H mice (97). In contrast, CS-exposed female B6C3F1 mice develop emphysema (143, 144). Mice have notable limitations, including less robust airway pathology, limited airflow obstruction despite parenchymal changes, and immune response patterns that differ from humans (145). Rat inflammatory profiles more closely resemble human disease, particularly regarding neutrophil recruitment, protease expression, and pronounced small airway remodeling and enhanced mucous cell metaplasia (146, 147).

The major advantage of mouse models is the development of animals with gene-targeted deletion that avail investigation of specific genes and molecular pathways in COPD pathogenesis. For example, AATD models are relevant given the established clinical link between AATD and early-onset emphysema (148). Furthermore, transgenic models have been invaluable in validating genetic insights from large population-based approaches. CS-exposed, Fam13a-deficient mice are protected against emphysema compared with WT controls, providing direct functional validation of this GWAS-identified risk locus (149).

Ferrets, sheep, and nonhuman primates (NHPs) have been used to overcome anatomical and physiological limitations of rodent models. Viral or bacterial challenges of smoke-exposed ferrets produce exaggerated inflammatory responses, worsened lung function, and prolonged recovery periods characteristic of COPD exacerbations (150). NHPs share approximately 93%–99% genetic homology with humans and possess comparable lung anatomy (151). Long-term CS exposure in NHPs produces emphysematous changes, airway remodeling, and mucus hyperproduction that closely parallels human disease (152). NHP models are particularly valuable for investigating age-related COPD aspects and evaluating biologics, which often show species-specific immune responses (153).

### Two-hit models.

Acute infections exacerbate COPD progression and mortality. In mice, CS exposure combined with bacterial LPS administration amplifies inflammatory responses and accelerates pathological changes compared with smoke/elastase alone (154, 155). Similarly, smoke exposure combined with viral infection (influenza, RSV, or rhinovirus) mirrors the virus-triggered exacerbations common in patients with COPD (156, 157). These approaches demonstrate synergistic effects on disease manifestation, dramatically reducing the experimental duration (158). Furthermore, integration of genetic factors with environmental exposures, as shown by combining AATD with CS exposure, recapitulates accelerated emphysema development (159).

## Pathomechanisms of COPD

Oxidative stress injury, resident cell senescence/death, and compromised wound repair are pivotal in COPD pathology and elicited by several mechanisms, including ROS production due to dysfunctional mitochondria, membrane-bound NADPH oxidases, and neutrophil-derived myeloperoxidase (MPO) (160). ROS also cause DNA damage, including bulky chemical adducts, methylated bases, oxidized purine and pyrimidine bases, abasic sites, and single- and double-strand breaks. Modifications of DNA bases that require base excision repair (BER) or nucleotide excision repair (NER) persist due to COPD-related defects in the genes responsible for these repair processes (161).

Pathological changes in COPD include compositional and quantitative changes in the ECM (162), collagen deposition, and reduced elastin (163). Regulatory proteins within the ECM, such as extracellular SOD3, are reduced in lungs of patients with COPD, causing remodeling of elastin, fibronectin, and collagen to increase airway resistance and reduce elastic recoil (162, 164). Furthermore, CS can increase MMP-12 activity in macrophages, disrupting the balance between proteolysis and antiproteolysis and inhibiting normal ECM repair (165–167).

Advances in single-cell technologies, particularly scRNA-seq, have dramatically expanded our understanding of COPD by identifying key cellular sources of pathobiology. These approaches offer unprecedented resolution of cell-type-specific transcriptional programs and state changes that underlie tissue remodeling, inflammation, and impaired regeneration. Critically, these technologies have contextualized findings from genetic and animal studies, pinpointing the specific cell types and complex intercellular interactions that contribute to COPD progression (Figure 2).

### Lung epithelial dysfunction in COPD.

Epithelial injury is central to COPD pathogenesis and occurs in AT2 pneumocytes, cells tasked with production of surfactant and serving as progenitors for alveolar type 1 (AT1) pneumocytes. scRNA-seq studies confirmed that AT2 cells exhibit two distinct functional states — one associated with homeostatic function and high surfactant production and another primed for epithelial repair (168–170). Progenitor AT2 cells give rise to a transitional AT2/AT1 population that is transcriptionally distinct from both lineages yet represents an intermediate cell state along the differentiation trajectory. In mice and organoid models, the intermediate state has been described as *Krt8^+^* alveolar differentiation intermediates (ADI) (171), or prealveolar type 1 transitional state (PATS) (172). A similar population in humans is enriched in chronic lung diseases, such as idiopathic pulmonary fibrosis, and are termed KRT5^–^KRT17^+^ or aberrant basaloid cells (173–175).

The characterization of novel terminal airway–enriched secretory cells (TASCs) and respiratory airway secretory (RAS) cells or collectively, terminal and respiratory bronchiolar secretory cells (TRB-SCs), is another important discovery (176, 177). This population are facultative progenitor cells restricted to terminal and respiratory bronchioles, marked by SCGB3A2 and SFTPB expression, and capable of differentiating into AT2 cells after injury. Lineage tracing in mice and organoid models show that these progenitors are regulated by hypoxia (178, 179), Notch, and Wnt (180) signaling, and significantly contribute to alveolar repair after injury (181, 182). Data also suggest that in COPD, SCGB3A2^+^ airway progenitor cells are depleted or undergo conversion to pathological cell states (183). Airway-derived AT2 cells have reduced regenerative capacity relative to native AT2 cells (183, 184). Collectively, changes in progenitor cell dynamics may reduce epithelial regenerative capacity both in airways and parenchyma in COPD. Single-cell studies have also revealed key insights into proximal airway biology relative to COPD.

### Immune, endothelial, and stromal cell dysfunction in COPD.

Single-cell profiling has delineated diverse macrophage populations, including alveolar, interstitial, and monocyte-derived subsets, each with specialized roles in lung immunity and remodeling. Notably, CS-induced macrophages originate from recruited monocytes and exhibit PRMT7-driven epigenetic modifications that enhance macrophage activation, monocyte recruitment, and chronic inflammation, ultimately exacerbating lung damage and emphysema (185). Single-cell approaches have shown expansion of cytotoxic CD8^+^ T cells, particularly TEMRA and tissue-resident memory (TRM) subsets, in COPD lungs (186). These IFN-γ–producing cells can impair AT2 cell function and differentiation of basal cells into TASCs/RAS/TRB-SC cells, altering epithelial regeneration (187). PRMT1 is also downregulated in pulmonary ECs in lungs from humans with COPD and mouse models, and PRMT1 loss exaggerates senescence and cell death via dysregulated NF-κB signaling (188). Single-cell profiling of fibroblasts shows upregulation of ECM remodeling genes that drives fibrosis and airway remodeling, while strongly influencing epithelial and endothelial behaviors through complex cell-cell signaling networks (189). GWAS further support this relationship between COPD and fibroblast biology, as suggested by COPD-associated SNPs in ECM signaling genes and by the presence of emphysema and connective tissue dysfunction in individuals with Mendelian forms of Loeys-Dietz syndrome type 4 and autosomal dominant cutis laxa (190–192).

### Multiomic approaches and spatial technologies.

Multimodal high-throughput technologies have yielded novel insights into COPD pathobiology. Integration of GWAS with scRNA-seq has enabled cell-type-specific mapping of susceptibility genes and identified enrichment in fibroblasts, smooth muscle cells, and epithelial and endothelial cells (193). Integration of transcriptomic, miRNA, and methylation data from lung tissue revealed that patients with similar clinical features, including severe airflow limitation and emphysema, can harbor distinct molecular endotypes (61). One cluster was marked by elevated B and T cell signatures and downregulation of secretory and ciliated cell genes, while the other lacked immune activation and showed distinct epithelial features.

Spatial technologies are beginning to fundamentally reshape our understanding of lung architecture and disease progression. While scRNA-seq has revealed discrete cellular states and differentiation trajectories, it lacks spatial context, an essential dimension in diseases like COPD (194, 195). Spatial approaches have investigated terminal airway narrowing, an early COPD pathological event that precedes development of emphysematous destruction (196). Airway narrowing is closely associated with loss of elastin fibers at alveolar attachments, which anchor alveoli to the small airways and maintain lung elasticity. Spatial imaging studies show macrophage and neutrophil accumulation around these alveolar attachments, where they are thought to contribute to elastin degradation. In parallel, adaptive immune cells, particularly CD8^+^ T cells, localize to terminal bronchioles and promote pathological remodeling, further exacerbating airflow limitation (196). This approach revealed that lymphoid follicles in emphysematous lung tissue exhibited a transcriptional profile marked by aberrant B cell activation, autoimmune-associated transcriptional regulators, and enhanced antigen presentation (197). Together, these findings highlight the spatially organized immune-structural interactions that drive early COPD progression.

### Spatial metabolomics.

Spatial metabolomics, particularly through matrix-assisted laser desorption/ionization (MALDI) imaging, represents another rapidly expanding frontier (198, 199). This technology enables in situ mapping of metabolites, lipids, and glycans at resolutions approaching the single-cell level. Metabolomics offers a direct readout of cellular function and biochemical activity, capturing real-time metabolic consequences of cellular states within intact tissue (200, 201). MALDI-based platforms now support profiling of complex molecular classes, including oxidative stress, that are implicated in COPD pathogenesis.

By integrating information on cell identity, location, and function, spatial metabolomics complements transcriptomic and proteomic data, facilitating generation of new multidimensional mechanistic hypotheses.

## Translational considerations

Currently, there are no curative or disease-modifying pharmacological therapies for COPD (202). While tobacco consumption and air pollution are leading COPD risk factors, they do not fully explain the risk of disease development or COPD heterogeneity. Genetics is an important contributor to COPD (203, 204), and advances in genotyping and sequencing technologies have made these discoveries possible. The effect size of each genetic signal is modest, limiting the efficacy of single-gene targeting approaches. But combinatorial analyses (i.e., PRS, genetics-genomics integration) invoking potentially targetable pathways are promising.

As is true for most chronic diseases, COPD has a long phase of pathobiological changes that can be detected before clinical diagnosis. Pre-COPD offers a unique opportunity to intervene before physiological reversibility and associated frailty and impaired lung function. Ongoing prospective studies will hopefully contribute to our understanding of early determinants of known comorbidities of patients with COPD, including hypertension, chronic kidney disease, irritable bowel syndrome, dementia, and cancer (205) (Figure 3).

Given the number of COPD-risk-associated loci (55), combinatorial therapy, rather than single agents, will likely be needed to address this disease effectively. In this context, RNA-based therapeutics such as antisense oligonucleotides (ASOs) offer advantages over traditional small-molecule-based drugs (206, 207). ASOs can be rapidly designed, qualify for expedited FDA approval, and allow early detection of off-target effects via transcriptomics (208–210). In 2023, the FDA approved tofersen, an ASO targeting *SOD1* mutations in amyotrophic lateral sclerosis (ALS) (211). Given the oligogenic nature of ALS, researchers are now pursuing personalized ASO cocktails tailored to each patient’s specific genetic mutations and relevant nongenetic disease drivers (212, 213).

Too few trials of candidate agents for COPD are being conducted, a clear reflection of suboptimal prioritization of informative trial design. While two previous trials (214) showed limited benefits from early treatment, clinical trials investigating pharmacological interventions in early COPD and pre-COPD are actively underway and hold promising potential. In addition, a vast library of well-characterized FDA-approved medications with potential to be repurposed for new COPD indications are in the pipeline, a strategy widely employed for malignancies and more recently COVID-19 (215, 216). The selection of agents, based on effects on known pathways together with creative trial designs, including antihypertensives (217) and antiglycemics, have been explored for efficacy in COPD trials (218). While pharmacological innovations are essential, preventive strategies aimed at lifestyle modifications such as smoking cessation, reduction of occupational exposures, promotion of physical activity and fitness, and improved diet and nutritional support are equally vital.

## Conclusions

The COPD-iNET symposium provided an important forum for experts from many fields to engage in discussions and knowledge exchange and underscored that the effects of COPD extend far beyond pulmonary pathology. As COPD associates with multiple comorbidities, mechanistic studies addressing COPD will have far-reaching impact beyond lung pathology and dysfunction. Issues raised in this Review represent the collective expertise and vision of a multidisciplinary group of investigators addressing COPD research and will serve as both a comprehensive resource for the current state of knowledge and a roadmap for future investigations. Our hope is that this worldwide effort in generating datasets that include underrepresented populations and integration of single-cell epigenetic, RNA, proteomic, and metabolomic datasets from patients and COPD models will help accelerate the development of novel therapeutic interventions for COPD. Sustained investment in collaborative network such as COPD-iNET, along with open data sharing and standardized phenotyping, will be crucial to translate these scientific advances into benefits for patients with COPD.

## Funding support

This work is the result of NIH funding, in whole or in part, and is subject to the NIH Public Access Policy. Through acceptance of this federal funding, the NIH has been given a right to make the work publicly available in PubMed Central.

- NIH grants R01HL155948, R21HL173512, and R01HL153400 (to MS).
- NIH grants R01HL141380 and R01HL179364 (to DC).
- NIH grants R01HL133135, R01HL152728, P01HL114501, and contract no. 75N92023D00011 (to EKS).
- NIH grant R01HL151107 (to VS).
- NIH grants R01HL168199, R01HL162813, R01HL153248, and R01HL135142 (to MHC).
- NIH grant R01HL068111 (to YT).
- NIH grants R01HL140839 and R01HL171213 (to YT and PC).
- NIH grants R01HL154343 and R01HL160008 (to EN).
- NIH grants R01HL146557, R01HL160939, and R01HL153375 (to PRT).
- NIH grant R01ES032081 (to BK).
- Department of Defense grant W81XWH2110414 (to BK).
- Department of Defense grants W81XWH2210629 and HT94252310034 (to MS).
- UK Research and Innovation Future Leaders Fellowship MR/X032914/1 (to RZJ).
- German Center for Lung Research (to AÖY and ML).
- German Research Foundation (DFG) grant 512453064 (to ML).
- Von Behring Röntgen Foundation grant 71_0011 (to ML).
- American Lung Association (to the COPD-iNET Symposium 2024).

## Figures and Tables

**Table 1:**
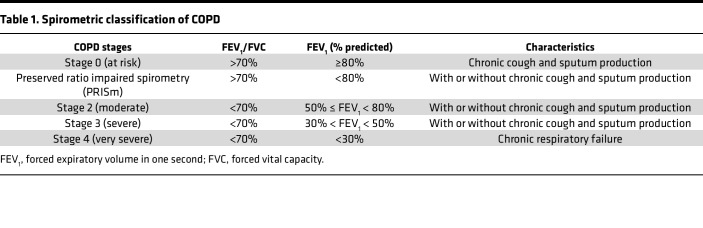
Spirometric classification of COPD

**Table 2:**
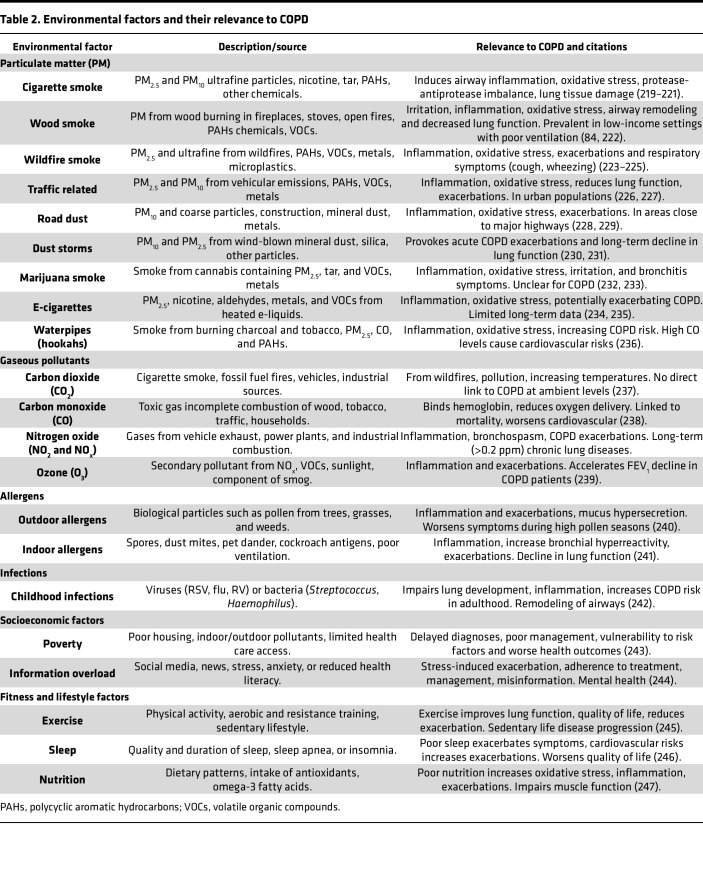
Environmental factors and their relevance to COPD

**Table 3:**
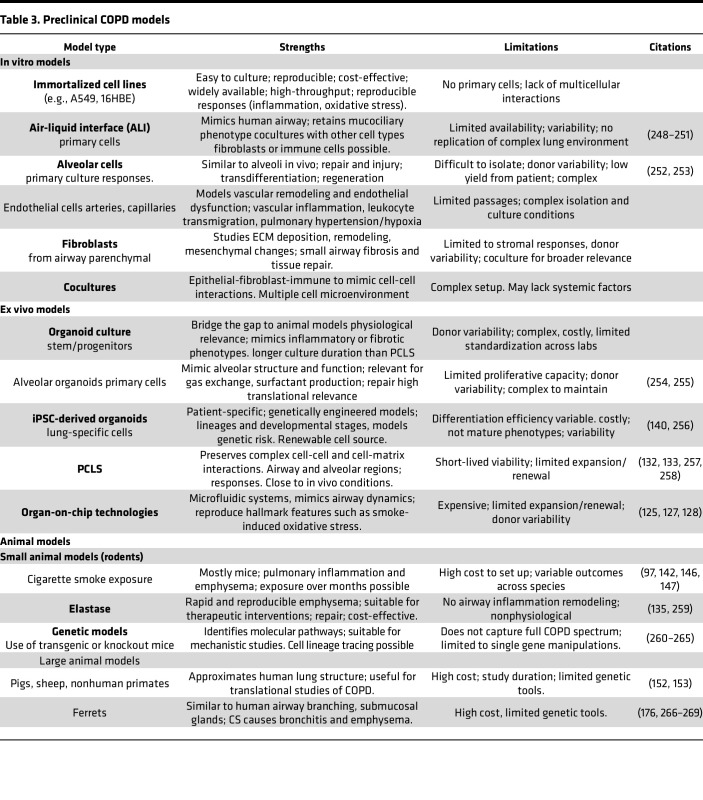
Preclinical COPD models

## Version 1 02/23/2026

Electronic publication
